# Impact of optional multidisciplinary tumor board meeting on the mortality of patients with gastrointestinal cancer: A retrospective observational study

**DOI:** 10.1002/cnr2.1373

**Published:** 2021-03-19

**Authors:** Mohammed Basendowah, Alaa M. Awlia, Hanin A. Alamoudi, Hala M. Ali Kanawi, Abdulaziz Saleem, Nadim Malibary, Hussam Hijazi, Mohammed Alfawaz, Anas H. Alzahrani

**Affiliations:** ^1^ Department of Surgery, Faculty of Medicine King Abdulaziz University Jeddah Kingdom of Saudi Arabia; ^2^ Faculty of Medicine King Abdulaziz University Jeddah Kingdom of Saudi Arabia; ^3^ Radiology Department, Radiation Oncology Unit, Faculty of Medicine King Abdulaziz University Jeddah Kingdom of Saudi Arabia; ^4^ Department of Medicine University of Jeddah Jeddah Kingdom of Saudi Arabia; ^5^ Clinical Research Education Program Icahn School of Medicine at Mount Sinai New York New York USA

**Keywords:** gastrointestinal cancer, mortality, multidisciplinary tumor board meeting, retrospective

## Abstract

**Background:**

Multidisciplinary tumor board meetings (MDTs) have shown a positive effect on patient care and play a role in the planning of care. However, there is limited evidence of the association between MDTs and patient mortality and in‐hospital morbidity for mixed cases of gastrointestinal (GI) cancer.

**Aim:**

To evaluate the influence of optional MDTs on care of patients with cancer to determine potential associations between MDTs and patient mortality and morbidity.

**Methods and results:**

This was a retrospective observational study at the referral center of King Abdulaziz University Hospital, Jeddah, Kingdom of Saudi Arabia. Among all adult patients diagnosed with GI cancer from January 2017 to June 2019, 130 patients were included. We categorized patients into two groups: 66 in the control group (non‐MDT) and 64 in the MDT group. The main outcome measure was overall mortality, measured by survival analysis. The follow‐up was 100% complete. Four patients in the MDT group and 13 in the non‐MDT group died (*P* = .04). The median follow‐up duration was 294 days (interquartile range [IQR], 140‐434) in the non‐MDT group compared with 176 days (IQR, 103‐466) in the MDT group (*P* = .20). There were no differences in intensive care unit or hospital length‐of‐stay or admission rates. The overall mortality at 2 years was 13% (95% confidence interval [CI], 0.06‐0.66) in the MDT group and 38% (95% CI, 0.10‐0.39) in the non‐MDT group (*P* = .08). The MDT group showed a 72% (adjusted hazard ratio [HR], 0.28; 95% CI, 0.08‐0.90; *P* = .03) decrease in mortality over time compared with the non‐MDT group.

**Conclusions:**

MDTs were associated with decreased mortality over time. Thus, MDTs have a positive influence on patient care by improving survival and should be incorporated into care.

## 1 INTRODUCTION

In 2018, the World Health Organization (WHO) reported that one in every six deaths is attributable to cancer and that the incidence of cancer is 18 million worldwide, with an estimated mortality of 9 million. In the same report, the estimated cancer mortality in the Kingdom of Saudi Arabia was reported to be 10 000.^1^ As the number of cancer cases increases, many challenges are raised about treatment modalities that suit patients' needs. Moreover, the rapid growth in treatment modalities has made the setting of a treatment plan more complex, and highly specialized personnel are required to provide optimal care to patients with cancer.^2^ One approach to treating cancer is personalized medicine, which replaces “one size fits all” models with individualized therapy.^3^ Another approach is patient‐centered care, which involves the provision of qualified, trustworthy, inter‐professional cancer care teams matched with patient needs, beliefs, and priorities, ensuring coordination between patient care teams and caregivers.^2^ Multidisciplinary management, which provides the advantage of having physicians from multiple specialties involved in care scheduling, is applied through multidisciplinary teams or multidisciplinary tumor board meetings (MDTs).^4^ An MDT is defined as a regularly scheduled meeting to review and set an integrated treatment plan for patients with cancer in the presence of experts such as surgeons, pathologists, radiologists, and medical and radiation oncologists.^5^

MDTs have been introduced over time in cancer care facilities across many countries, and participation has been recommended to ensure prompt and adequate care by a variety of professionals.^6^ MDTs have shown a positive effect on patient care and play a role in the planning of care.^7^ For instance, 26% of patients with colorectal cancer had changes in their treatment plans after an MDT.^8^ In another study, 29% of patients presenting with primary rectal cancer had changes in their treatments after their cases were discussed in an MDT.^9^ Additionally, MDTs facilitated preoperative staging in 96% of patients with rectal cancer.^10^ A systematic review showed that patients who were discussed at an MDT had a more accurate diagnosis and complete preoperative staging. The authors reported that the diagnostic reports changed in 4% to 35% of cases.^11^ Patients presented to an MDT have better adherence to disease‐specific guidelines and shorter time from diagnosis to treatment.^11^, ^12^

Patients who were discussed at an MDT had better survival than those who were not.^13^ In another study, the 3‐year survival rate in patients with advanced colorectal cancer was 66% in the MDT group vs 58% in the non‐MDT group.^14^ The 1‐year survival rate in patients with non‐small cell lung cancer discussed at an MDT was 33% compared with 18% in those who were not.^13^ Previous literature suggests that further evaluation should be based on the quality and effectiveness of an MDT on treatment plans.^9^, ^11^, ^15^

Despite the benefits of MDTs, there is limited evidence of the association between MDTs and mortality and in‐hospital morbidity for mixed cases of gastrointestinal (GI) cancer.^11^, ^15^ In the absence of evidence, it is unclear whether optional MDTs should become a mandatory standard of care. Thus, the aim of this study was to measure the impact of an optional MDT on patients' mortality and in‐hospital morbidity. We also aimed to examine disease‐specific mortality among patients with colorectal, stomach, pancreatic, and hepatobiliary cancers.

## 2 METHODS

### 2.1 Formation of an MDT

An MDT was started in our center in January 2017. Our MDT includes the disciplines of medical oncology, gastroenterology, hepatology, surgery, diagnostic and interventional radiology, radiation oncology, and pathology. The MDT is optional, and the decision on whether a patient's case should be presented in the MDT is made by the primary treating physician.

### 2.2 Setting and design

This retrospective observational study was conducted at King Abdulaziz University Hospital (KAUH), Jeddah, Kingdom of Saudi Arabia. Using the electronic health information system of KAUH, we reviewed all patients who were diagnosed with GI cancer from January 2017 to June 2019. We excluded pediatric, obstetrics/gynecology, or non‐GI patients. Our team collected demographic, clinical, treatment plan, and death data. Age‐adjusted Charlson Comorbidity Index (ACCI) was calculated to account for baseline patient morbidities. The research team followed up patients on a monthly basis in the outpatient clinics of general surgery, oncology, and gastroenterology. At the end of the study, patients' journals and death records were reviewed to confirm if patients had died. This study was approved by the Institutional Review Board at King Abdulaziz University (KAU), Jeddah, Kingdom of Saudi Arabia (IRB no. 473‐19). We followed the Strengthening the Reporting of Observational Studies in Epidemiology (STROBE) guidelines for reporting our findings.

### 2.3 Variables and outcomes

Cancer recurrence and metastasis were defined as the prevalence of cancer occurrence before the last event of current cancer that was included in our final analysis as a baseline characteristic. For the sake of analyzing the outcomes, we combined colon and rectal cancer into the colorectal cancer (CRC) category and combined liver cancer, cholangiocarcinoma, and pancreatic cancer into the hepatobiliary and pancreatic (HBP) cancer category. We did not include patients with small bowel or esophageal cancer as we only had a total of three patients with these conditions and all of them were discussed in the MDT, with no patients in the control group (non‐MDT). We considered patients who were not treated in our center as missing, and thus, we dropped them from our analysis. We categorized patients into two groups: the MDT group, if patients were discussed in an MDT, and the control group (non‐MDT), if patients were not discussed in an MDT. The primary outcome was overall mortality, and the secondary outcome was disease‐specific mortality for patients with stomach cancer, HBP cancer, and CRC.

### 2.4 Statistical analysis

Demographic and clinical characteristics were compared between the two groups using a two‐way *t*‐test if the data were normally distributed or the Mann‐Whitney test in case of non‐normally distributed data. Fisher's exact test or the chi‐squared test was used for cross tabulation of data. We defined the time to event as the time from diagnosis to death or end of the study. We constructed unadjusted Kaplan‐Meier estimates to describe the failure function in comparing the non‐MDT and MDT groups, and the log‐rank and Wilcoxon tests were applied to test equality. Adjusted failure‐function curves were constructed to describe the time to death between groups after adjusting for other factors. Univariable and multivariable Cox proportional hazards analyses were used to identify the independent factors for overall mortality. Multivariable Cox proportional hazards analysis was stratified by cancer type for subgroup analysis. Covariates in the models included male sex, BMI, nationality, ACCI score, and history of cancer recurrence, which were selected based on clinical relevance, prior literature, and the guidance of likelihood‐ratio tests to achieve model parsimony. A *P* value of <.05 was considered statistically significant. Analyses were conducted using Stata 14 statistical software (StataCorp, College Station, TX, USA).

## 3 RESULTS

### 3.1 Patients' demographic and clinical data

Over the study period, a total of 130 patients were included. Sixty‐six patients were in the non‐MDT group and 64 patients were in the MDT group. Patients' demographics and clinical data are summarized in Table 1. The non‐MDT group had more patients with CRC (55, 83%) than the MDT group (41, 64%) (*P* = .02). In contrast, the non‐MDT group had fewer patients with HBP cancer (4, 6%) than the MDT group (18, 28%) (*P* < .01). No significant differences were observed between the two groups with regard to age, body mass index (BMI), nationality, sex, history of cancer recurrence, ACCI score, or metastasis. Tables 2 and 3 shows no significant difference in treatment plans between groups, except that 21 (33%) patients in the non‐MDT group were treated by a combination of chemotherapy, radiotherapy, and surgery compared with eight (13%) in the MDT group (*P* = .01). Six (9%) patients in the MDT group were treated with other therapies, for example, portal vein (PV) embolization, radio‐frequency ablation (RFA), and imatinib, compared with one patient in the non‐MDT group (*P* = .12).

**TABLE 1:** Demographic and clinical data

	Non‐MDT = 66	MDT = 64	*P* value
Age, mean (SD)	55.7 (10.6)	57.1 (13.1)	.50
BMI, mean (SD)	26.5 (6.1)	25.86 (6.2)	.56
Saudi nationality, %	30 (45%)	29 (45%)	.99
Male, %	32 (48%)	30 (46%)	.86
History of cancer recurrence, %	4 (6%)	7 (11%)	.36
ACCI score			
1‐3, %	18 (27%)	12 (19%)	.68
4‐6, %	30 (46%)	31 (48%)	
7‐9, %	16 (24%)	19 (30%)	
10‐12, %	2 (3%)	3 (3%)	
Diagnosis			
Stomach cancer, %	7 (11%)	5 (8%)	.76
Colorectal cancer, %	55 (83%)	41 (64%)	.02
Hepatobiliary and pancreatic cancer, %	4 (6%)	18 (28%)	<.01
History of cancer metastatic, %	26 (40%)	24 (38%)	.86

Abbreviations: ACCI, age‐adjusted Charlson Comorbidity Index; BMI, body mass index; MDT, multidisciplinary tumor board meeting.

**TABLE 2:** Treatment plans for patients in the MDT (multidisciplinary tumor board meeting) and non‐MDT groups (overall treatment plans)

	Non‐MDT = 66	MDT = 64	*P* value
Adjuvant chemotherapy, %	19 (29%)	14 (22%)	.42
Neoadjuvant chemotherapy, %	39 (59%)	38 (59%)	.99
Adjuvant radiotherapy, %	4 (6%)	1 (1.5%)	.36
Neoadjuvant radiotherapy, %	24 (36%)	15 (23%)	.13

**TABLE 3:** Treatment plans for patients in the MDT (multidisciplinary tumor board meeting) and non‐MDT groups (details of treatment plans)

	Non‐MDT = 66	MDT = 64	*P* value
Surgery alone, %	6 (9%)	7 (11%)	.99
Chemotherapy alone	12 (19%)	18 (28%)	.30
Radiotherapy alone	2 (3%)	2 (3%)	.99
Surgery and chemotherapy	16 (25%)	19 (30%)	.69
Surgery and radiotherapy	1 (1%)	0 (0%)	.99
Chemotherapy, radiotherapy, and surgery	21 (33%)	8 (13%)	.06
Chemotherapy and radiotherapy	5 (8%)	4 (6%)	.99
Other therapy	1 (1.5%)	6 (9%)	.12
Palliative	14 (21%)	10 (16%)	.50

### 3.2 In‐hospital morbidity

There were no differences in intensive care unit (ICU) admission and readmission after surgery between the two groups. The median follow‐up duration was 294 days (interquartile range [IQR], 140‐434) in the non‐MDT group compared with 176 days (IQR, 103‐466) in the MDT group (*P* = .20) (Table 4). There were no significant differences between the groups with regard to hospital length of stay, ICU length of stay, or time from diagnosis to surgery (Table 4).

**TABLE 4:** In‐hospital morbidities

	Non‐MDT = 66	MDT = 64	*P* value
Hospital length of stay, median (IQR)	15 (10‐22)	10 (8‐16)	.06
Readmission	8 (12%)	8 (12%)	.99
ICU admission	22 (33%)	18 (28%)	.57
ICU length of stay, median (IQR)	2 (1‐3)	2 (1‐3)	.97
Diagnosis to surgery, median (IQR)	93 (13‐157)	21 (12‐152)	.36
Follow‐up time, median (IQR)	294 (140‐434)	176 (103‐466)	.20

Abbreviations: ICU, intensive care unit; IQR, interquartile range; MDT, multidisciplinary tumor board meeting.

### 3.3 Overall mortality and specific mortality

**FIGURE 1:**
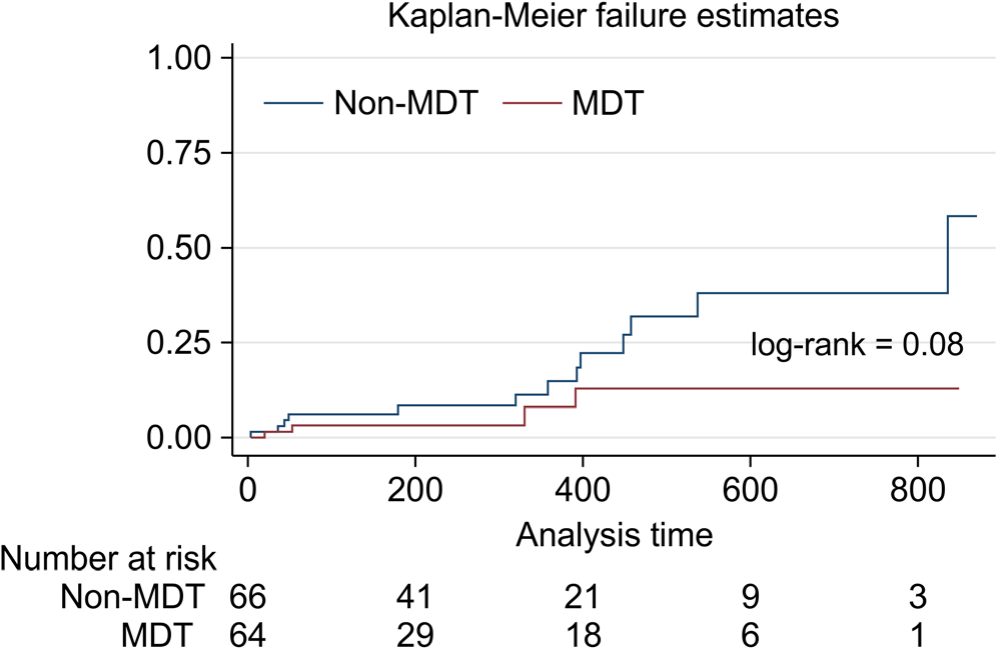
Kaplan‐Meier failure estimate graph showing mortality in the non‐MDT group (blue) vs MDT group (red). No significant differences were found in overall mortality at 6 months, 1 year, and 2 years: 3% (95% CI, 0.01‐0.13), 8% (95% CI, 0.05‐0.26), and 13% (95% CI, 0.04‐0.34) in the MDT group and 8% (95% CI, 0.04‐0.19), 15% (95% CI, 0.06‐0.30), and 38% (95% CI, 0.22‐0.60) in the non‐MDT group, respectively (*P* = .08). MDT, multidisciplinary tumor board meeting. Log‐rank test used to test the difference between non‐MDT and MDT survival curve. *P* value <.05 considered as statistically significant

**FIGURE 2:**
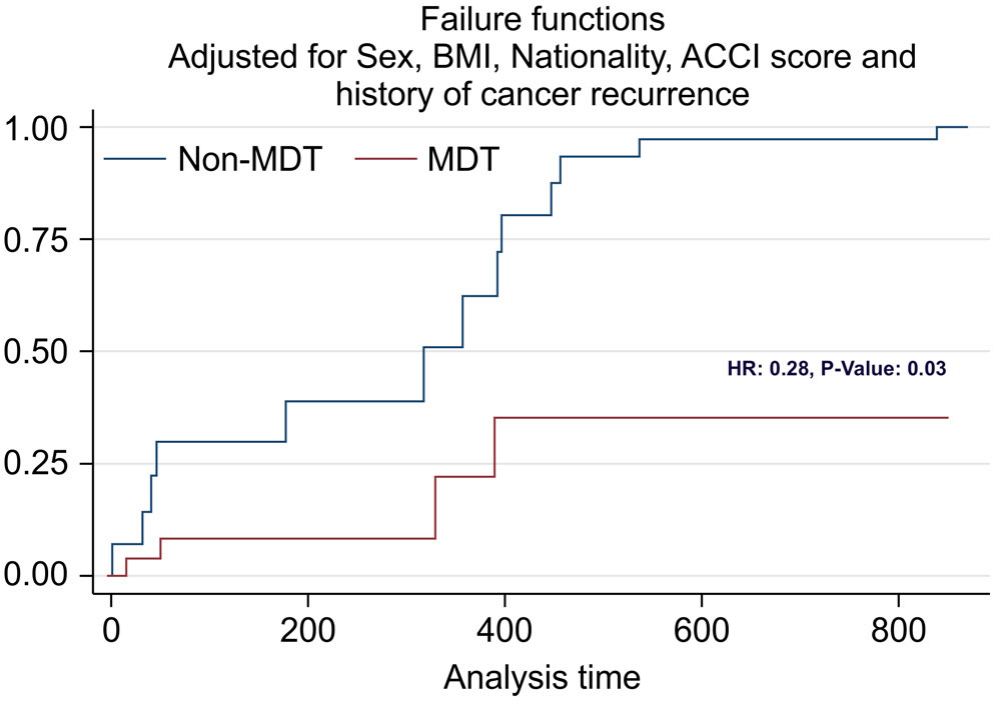
Adjusted Kaplan‐Meier failure estimate graph showing mortality in the non‐MDT (blue) vs MDT group (red). When adjusting for other factors, our model showed that patients who were discussed at an MDT had a 72% decrease in mortality risk than those who were not (adjusted HR, 0.28; 95% CI, 0.08‐0.90; *P* = .03). Hazard ratio (HR) used to estimate the adjusted difference between two groups. *P* value <.05 considered as statistically significant

Over the study period, the follow‐up was 100% complete, with no patients censored. Four patients (6%) in the MDT group died compared with 13 (20%) in the non‐MDT group (*P* = .04). No significant differences were found in overall mortality at 6 months, 1 year, and 2 years: 3% (95% CI, 0.01‐0.13), 8% (95% CI, 0.05‐0.26), and 13% (95% CI, 0.04‐0.34) in the MDT group and 8% (95% CI, 0.04‐0.19), 15% (95% CI, 0.06‐0.30), and 38% (95% CI, 0.22‐0.60) in the non‐MDT group, respectively (*P* = .08) (Figure 1). However, on adjusting for other factors, our model showed that patients who were discussed at an MDT had a 72% decrease in mortality risk than those who were not (adjusted HR, 0.28; 95% CI, 0.08‐0.90; *P* = .03) (Table 5 and Figure 2). Multivariable Cox regression showed that with every unit of increase in BMI, there was a 12% decrease in overall mortality risk over time (adjusted hazard ratio [HR], 0.88; 95% confidence interval [CI], 0.79‐0.97; *P* < 0.01). In the subgroup analyses, illustrated in Table 6, the mortality rate decreased over time by 71% (adjusted HR, 0.29; 95% CI, 0.08‐0.99; *P* = .048), 71% (adjusted HR, 0.29; 95% CI, 0.09‐0.96; *P* = .043), and 73% (adjusted HR, 0.27; 95% CI, 0.07‐0.98; *P* = .047) for stomach cancer, CRC, and HBP cancer, respectively.

**TABLE 5:** Univariable and multivariable Cox regression

Variables	Univariable HR (CI)	PV embolization	Multivariable HR (CI)	*P* value
Male	1.63 (0.63‐4.25)	0.32	2.13 (0.80‐5.69)	.13
BMI	0.90 (0.81‐0.98)	0.03*	0.88 (0.79‐0.97)	<.01
Saudi nationality	0.89 (0.33‐2.34)	0.80	0.99 (0.36‐2.68)	.98
ACCI score	1.61 (0.87‐2.98)	0.12	1.72 (0.87‐3.40)	.12
History of cancer recurrence	0.70 (0.09‐5.24)	0.72	1.19 (0.13‐10.98)	.88
Tumor board	0.38 (0.12‐1.18)	0.09	0.28 (0.08‐0.90)	.03

**TABLE 6:** Multivariable Cox regression for cancer types

		Multivariable HR (CI)	*P* value
Model 1	Stomach cancer		
	Tumor board	0.29 (0.08‐0.99)	.048
Model 2	Colorectal cancer		
	Tumor board	0.29 (0.09‐0.96)	.043
Model 3	Hepatobiliary and pancreatic		
	Tumor board	0.27 (0.07‐0.98)	.047

Abbreviations: ACCI, age‐adjusted Charlson Comorbidity Index; BMI, body mass index; CI, confidence interval; HR, hazard ratio; PV, portal vein.

## 4 DISCUSSION

This study determined the association between optional MDTs and the risk of mortality by comparing patients with GI cancer who were discussed at an MDT vs those who were not. We found no difference in unadjusted survival between the MDT and non‐MDT groups. However, we found that an MDT was associated with decreased overall mortality in patients with mixed GI cancer and with decreased mortality in patients with stomach cancer, HBP cancer, and CRC after adjusting for other factors.

This pattern of improved overall survival has been noticed in GI and non‐GI cancers. For instance, Bydder et al showed that the 1‐year survival rate for lung cancer was 33% for MDT patients compared with 18% for non‐MDT patients.^13^ Similarly, survival improvement has been found in other cancer types, including gynecological, breast, urological, and head and neck cancers.^11^ In line with our results, MDT patients showed a 72% decrease in mortality rate compared with non‐MDT patients, after adjusting for other factors. A systematic review summarized that the improvement in survival resulted from better‐chosen treatment plans.^11^ This suggests that MDTs should be a part of the standard of care and not limited to physician choice.

Our finding of improvements in specific mortality associated with stomach cancer, CRC, and HBP cancer (71%‐73%) is similar to the results of other studies. A study conducted in patients with rectal cancer after the inception of MDT showed that MDT patients had lower postoperative mortality (5%) than non‐MDT patients (9%).^16^ Furthermore, a study conducted among CRC patients showed that MDT was an independent factor for survival, as the 3‐year survival post‐MDT establishment was 66% compared with 58% in non‐MDT patients.^14^ Another study assessed the evolution of colorectal cancer treatment over the years in a single center, where the 3‐year overall survival after the initiation of a multidisciplinary colorectal cancer center was dramatically increased, to 82% from 65%.^17^ Several previous studies have looked into survival among patients with hepatocellular carcinoma before and after MDT establishment,^18^, ^19^ and all found improved survival post‐MDT. One of these studies found a 28% reduction in mortality among MDT‐discussed cases compared with non‐MDT‐discussed cases.^18^

According to our results, the prevalence of CRC was lower and that of HBP cancer was higher in the MDT group than in the non‐MDT group. This might be explained by the fact that CRC is ranked as the most common cancer in men and the third most common cancer in women in Saudi Arabia, as reported by the WHO; well‐defined guidelines are available for CRC, making physicians more competent in managing this type of cancer. In contrast, HBP malignancies are less common and tend to present at more advanced stages; thus, physicians need to present such patients to the MDT to obtain more insight from MDT members. Nonetheless, we found that patients with CRC and HBP cancer who were presented in the MDT had decreased mortality. This might be due to the effectiveness of an MDT for common cancers such as CRC and rare cancers such as HBP cancer.

Unsurprisingly, we found that BMI had a role in the mortality of patients with cancer; mortality decreased by 12% with every 1 unit increase in BMI. This indicates that patients who are generally fit and healthy survive longer. Our finding is in line with that of a previous study that compared CRC patients with a normal BMI with those with changes in BMI; the study showed that for stage III disease, lower BMI was associated with an 87% increase in mortality. Furthermore, a protective effect was noted for stage IV disease, as there was a 42% decrease in mortality among those with a higher BMI.^20^ Other evidence supporting our finding comes from Li et al, who found that for each 5% decrease in BMI, there was a 27% increase in overall mortality; they concluded that increased mortality was linked to lower BMI.^21^ In contrast, Shaukat et al's study on CRC mortality found that a higher BMI was related to increased mortality.^22^ These findings may guide the MDT and improve a patient's general health by the inclusion of not only physicians but also nurses and nutritionists in the MDT.^23^

Morbidity was measured by comparing ICU admission, hospital length of stay, readmission rate, and follow‐up time. Both groups showed similar results in terms of morbidity. With regard to follow‐up time, Chang et al studied the effect of an MDT on patients with hepatocellular carcinoma and found that the median follow‐up time was increased significantly from 4.5 months to 9.5 months after its implementation.^24^ Freeman et al's research focusing on thoracic cancer found that MDT patients showed a significantly shorter time from diagnosis to treatment.^12^, ^25^ Another study conducted on pancreatic and other upper GI malignancies concluded that presenting cases at the MDT resulted in a 25% change in the treatment plan.^26^ Additionally, Gardner et al studied the effect of MDT clinics on treatment accessibility in patients with pancreatic cancer, revealing a shorter period of time from diagnostic biopsy to the initiation of therapy (by 22 days) than that in non‐MDT clinics.^27^ Moreover, for hepatocellular carcinoma, the median time to treatment was shortened from 5.3 to 2.3 months in MDT clinics.^19^ In contrast, our study included a variety of GI cancers (colon, rectal, stomach, pancreatic, and hepatobiliary cancers); with regard to the time from diagnosis to surgery, our study showed a median duration of 93 days (IQR, 13‐157) in the non‐MDT group in contrast to 21 days (IQR, 12‐152) in the MDT group. This difference was not statistically significant. Furthermore, the research team conducted a post hoc analysis using Poisson regression with and without a zero‐inflated model to test if there was a real difference between groups. Neither univariable nor multivariable analysis showed statistically significant differences. Although there may be a true difference between the MDT and non‐MDT groups for every specific cancer type with regard to time from diagnosis to surgery, considering our study aim and design, we did not collect detailed data about the time from diagnosis to treatment and we had limited data about the time from diagnosis to surgery. Thus, we did not observe a true difference between the groups in our analysis. Another possible explanation is that surgeons who presented their cases to the MDT made decisions to operate without neoadjuvant chemotherapy and/or neoadjuvant radiotherapy, believing that such decisions were supported by other disciplines. For example, for right colon cancer without metastasis, they might go directly to surgery, in the confidence that the MDT will not alter the treatment plan.

We expected the primary physicians of non‐MDT groups to follow the formal guidelines without consulting other members of other disciplines. Thus, we found that the combination of chemotherapy, radiotherapy, and surgery was used more frequently in the non‐MDT group. However, the MDT group had more tumor‐specific therapy, including PV embolization, RFA, and imatinib treatment. The consideration of more advanced treatment options is likely to be the result of a case‐by‐case discussion at an MDT by highly specialized physicians from various specialties.

There are many examples of patients who were discussed in MDTs who received more advanced treatment than those who were not discussed in MDTs. As reported by Morris et al, MDT discussions resulted in a 22% increase in the usage of radiotherapy in patients with breast cancer.^28^ Boniface et al studied esophageal and gastric tumor discussions in MDTs and found that most participants had a change in the pathological diagnosis.^29^ Another example comes from the field of head and neck surgery; Wheless et al found that MDTs changed the diagnosis, treatment plans, and staging in 27% of the study population, adding more multimodality care.^30^ This finding was supported by those of Kurpad et al, who found a 38% change in diagnosis and/or treatment of patients diagnosed with urological malignancies.^5^ Greer et al also studied the impact of an MDT on gynecological malignancies; they reported that the pathological diagnosis was changed in 27% of cases, with 74% of those having changes in the management, with an overall change of 20% in the management plan of the presented pathology reports. Another perspective was that radiological presentations, a new diagnosis, or upstaging was modified in 10% of the presented cases.^31^ Another study conducted among 149 patients with GI malignancies, one‐third with pancreaticobiliary cancer, another third with liver cancer, and the remaining with other GI cancers, found that 36% of all cases involved changes in the treatment plan.^32^ Pawlik et al investigated the role of multidisciplinary clinics in managing pancreatic malignancy: 23.6% and 18.7% of cases discussed involved changes in their management plans and radiological interpretations, respectively.^33^ Among patients with rectal cancer, restaging was performed in 7% of patients post‐MDT.^34^

This study has several strengths. Our literature search showed that no previous study had assessed the effectiveness of an MDT on patients' mortality and morbidities in our region. Another strength is that we measured the baseline comorbidities by using the ACCI and included this in our analysis. Our control group (non‐MDT) matched the period of an MDT to avoid the history bias, so both groups received the same up‐to‐date available treatment in our center. Lastly, this study had a long follow‐up time.

There are other questions that our study was not designed to answer. First, we are aware that the mortality and prognosis in GI cancer differ according to the histopathological diagnosis and radiological staging, but we decided to look into the effectiveness of an MDT in general. For that, we did not collect data on histopathology, radiological staging, performance status, albumin, or lactate dehydrogenase. Second, the change in treatment plans due to an MDT is well documented in the literature. However, we did not collect any data about the change in treatment plans, and our research team decided to focus on more meaningful clinical outcomes that reflect the patients' general health. Third, in our center, MDT discussions are optional, which places the decision of presenting the patients' cases in the hands of the primary treating physician. This, along with the nature of our study design, might introduce selection bias. Despite the fact that our center is considered one of the largest centers for treating cancer in the city of Jeddah, Saudi Arabia, this study is a single‐center study with a small sample size, which might affect the generalizability of our findings and limit our conclusions to similar centers. To address these limitations in our study design, further research using a multicenter prospective study that includes a large sample size and homogeneous cohort with cancer‐specific data (eg, data on radiological staging, performance status, albumin, and lactate dehydrogenase) is recommended.

## 5 CONCLUSIONS

This study revealed that MDTs had a significant role in decreasing mortality, with no effect on morbidity. This may have been because of the well‐structured and integrated management plan provided to MDT patients, making them and their treatment algorithm well known to the oncology services in the hospital. MDTs have a meaningful positive influence on patient care by improving survival and should be incorporated into the standard care of patients with cancer. We recommend including other disciplines in the MDT, for example, nurses and nutritionists, to improve patients' general health before treatment. A further multicenter prospective study that includes cancer‐specific data (eg, data on radiological staging) is recommended.

## CONFLICT OF INTEREST

The authors have stated explicitly that there are no conflicts of interest in connection with this article.

## AUTHOR CONTRIBUTIONS

All authors had full access to the data in the study and take responsibility for the integrity of the data and the accuracy of the data analysis. *Conceptualization*, M.B., A.A., H.A., H.K., A.S., N.M., H.H.; *Data Curation*, M.B., A.A., H.A., H.K.; *Formal Analysis*, M.B., A.A., A.S., N.M., H.H., M.A.; *Investigation*, M.B., A.A.; *Methodology*, M.B., A.A., H.A., H.K., A.S., N.M., H.H.; *Project Administration*, M.B., A.A., H.A., H.K., H.H.; *Resources*, M.B., A.A., H.A., H.K.; *Software*, M.B.; *Supervision*, M.B.; *Validation*, M.B.; *Visualization*, M.B.; *Writing‐Original Draft*, M.B., A.A., H.A., H.K., A.S., N.M., H.H., M.A.; *Writing‐Review & Editing*, M.B., A.A., H.A., H.K., A.S., N.M., H.H., M.A.

## ETHICAL STATEMENT

This study was approved by the Institutional Review Board at King Abdulaziz University (KAU), Jeddah, Kingdom of Saudi Arabia (IRB no. 473‐19). Informed consent was waived because of the retrospective design of the study.

## Data Availability

Data available on request due to privacy/ethical restrictions.
